# Impact of music performance anxiety on cardiovascular blood pressure responses, autonomic tone and baroreceptor sensitivity to a western classical music piano-concert

**DOI:** 10.3389/fnins.2023.1213117

**Published:** 2023-07-19

**Authors:** Juan Ángel Moreno-Gutiérrez, Carmen de Rojas Leal, Manuel Víctor López-González, Alvaro Chao-Écija, Marc Stefan Dawid-Milner

**Affiliations:** ^1^School of Medicine, Autonomic Nervous System Unit, CIMES, University of Málaga, Malaga, Spain; ^2^Department of Neurology, Hospital Universitario Virgen de La Victoria, Malaga, Spain; ^3^Biomedical Research Institute of Málaga (IBIMA), Málaga, Spain

**Keywords:** music performance anxiety (MPA), sympathetic nervous system, parasympathetic nervous system, Wigner-Ville analysis (W-V), baroreceptor sensibility, autonomic nervous system, piano performance, heart rate variability

## Abstract

**Introduction:**

Music Performance Anxiety (MPA) is a prevalent condition among musicians that can manifest both psychologically and physiologically, leading to impaired musical performance. Physiologically, MPA is characterized by excessive muscular and/or autonomic tone. This study focuses on the cardiovascular blood pressure responses, autonomic tone and baroreceptor sensitivity changes that occur during musical performance due to MPA.

**Methods:**

Six professional pianists perform a piece for piano written only for the left hand by Alexander Scriabin. The following parameters have been studied during the performance: ECG, non-invasive beat to beat continuous arterial blood pressure and skin conductance. Sympathetic and parasympathetic autonomic flow was studied with Wigner-Ville analysis (W-V) from R-R ECG variability, and baroreceptor sensitivity with the Continuous Wavelet Transform (CWT).

**Results:**

During the concert a significant increase of heart rate, systolic, mean and diastolic arterial pressure were observed. No significant differences were found in skin conductance. The W-V analysis, which studies frequency changes in the time domain, shows a significant increase of sympathetic flow and a decrease of parasympathetic flow during the concert which is associated with a significant decrease in sympathetic and vagal baroreceptor sensitivity.

**Discussion:**

The study of cardiac variability using the Wigner-Ville analysis may be a suitable method to assess the autonomic response in the context of MPA, and could be used as biofeedback in personalized multimodal treatments.

## Introduction

Music is a cultural product which is inherent to the lifestyle of humanity over time. For musicians, to make a difference with respect to the rest of the performers, it is imperative to achieve a level of performance that meets the expectations of themselves and their audience. Meeting these requirements may generate performance-related anxiety or Music Performance Anxiety (MPA), in most cases causing a worsening of musical performance (Yoshie et al., 2009a). This circumstance arises when there is a perception of subjective evaluation, which may stem from an internal assessment or, more commonly, from an external source, like during public performance. For this reason, it is included in the DSM-V classification as a sub-type of social phobia (Spahn et al., 2021a,b; American Psychiatric Association, 2022; Guyon et al., 2022).

The prevalence of MPA varies across different studies, but it is generally a widespread problem among interpreters. Fernhol estimates the prevalence to be between 16.5 and 60% (Fernholz et al., 2019). Additionally, Spahn found that about 90% of orchestral musicians in Germany suffer from anxiety and stress (Spahn, 2015). A survey of 2,212 musicians revealed that before playing in public, more than 20% used beta-blockers to eliminate the vegetative symptoms associated with this picture (Yoshie et al., 2009a).

The MPA can manifests as both psychological stress or physiological changes and, it can occur from the preparation of a performance to late periods after the performance itself (Spahn et al., 2021a).

Psychologically, symptoms can be cognitive on one hand, displayed as a heightened state of alertness, with a greater focus on the possibility of making mistakes rather than on the interpretation itself, accelerated tempo due to altered time perception, increased attention to irrelevant tasks, loss of attention, self-awareness and sense of control (Yoshie et al., 2009a; Chang-Arana et al., 2022; Guyon et al., 2022; Sokoli et al., 2022); or, on the other hand, can be affective with negative thoughts and feelings that could even lead to depression (Sickert et al., 2022).

Physiologically, the MPA manifests itself in two different ways. As an increase in muscle tone and /or as an increase in autonomic tone. As suggested by Yoshie et al. (2009b), anxiety could increase the discharge frequency of the corticospinal tract, facilitating an exaggerated motor response secondary to stimulation of the amygdala, which in turn activates the basal and thalamic ganglia. This could increase motor flow increasing the excitability of the motor cortex, generating a greater tone, with electromyographic overactivity in the muscles of the upper limb (Yoshie et al., 2009a). This increased muscle activity can result in a greater finger pressure on the keyboard leading to a loss of fine motor control. This loss of control results in a deficit of flexor-extensor motor coordination due to over-activation of the antagonistic muscles of the upper limb. The combination of increased muscle tone and strength with a loss of coordination can generate a sense of heightened tension for the interpreter, which is a hallmark of MPA (Yoshie et al., 2009a; Kotani and Furuya, 2018).

MPA is also associated with an exaggerated autonomic tone (Chang-Arana et al., 2023), which increases sympathetic adrenergic activation raising heart rate, arterial blood pressure (not continuously measured), and the rate and respiratory flows (Guyon et al., 2020a,b). Sympathetic cholinergic activation increases sweating and therefore reduces skin resistance (Nakahara et al., 2010). Autonomic overresponse of MPA is greater in a public situation than in a solitary study of the instrument or voice (Yoshie et al., 2009a,b). The neurophysiological explanation of these responses involves understanding the functional organization of the autonomic nervous system (ANS).

The neurovegetative response to physical or psychological stress involves the supraencephalic activation of the brainstem nuclei involved in the control of sympathetic activity. The main structure in charge of relating emotional states and anxieties is the so-called “Anterior Limbic Circuit” made up of the insular cortex, the anterior cingulate cortex and the amygdala. This circuit interconnects with the prefrontal cortex, which regulates emotional behaviour and decision (Shouman and Benarroch, 2023) through projections acting at brainstem-pontine levels. The nucleus of the solitary tract, the rostro ventrolateral medulla and the parabrachial nucleus are involved in central anxiety responses modulating neurovegetative medullary reflexes, such as the baroreceptor reflex, chemoreceptor reflex, mechanical pulmonary reflexes, and cardiac reflexes that control blood pressure and heart rate (Silva-Carvalho et al., 1995; López-González et al., 2018; González-García et al., 2023).

As the stressful conditions increase or the psycho-cognitive influence is less inhibited, the stimulation of the hypothalamic regions increases markedly, thus generating a massive sympathetic discharge (Oubaid, 2023). This phenomenon is known as a defence or flight reaction since it provides the necessary autonomic tone to carry out behavioural actions in the face of imminent danger (Díaz-Casares et al., 2012; López-González et al., 2018). Simultaneously, this increase in tonic outflow translates into an increase in motor flow in the cortical spinal pathway, which also increases muscle tone and strength, facilitating the defence response (Nakahara et al., 2010). Increased sympathetic adrenergic and cholinergic flow, together with increased muscle tone and strength are typical effects of MPA (Jha et al., 2022).

MPA heart rate changes during piano performance are well known, but no information exists about possible continuous blood pressure changes. Thus, the main objective of this work was to record beat to beat arterial blood pressure during MPA piano performance and to analyse the possible changes and the origin of these changes.

## Materials and methods

The study was carried out at the CIMES Autonomic Nervous System Laboratory under controlled conditions of temperature (24° degrees) and relative humidity (30%).

9 young professional classic pianists participated as volunteers (5 females, 4 males). They were all right-handed and had an average training experience of 14.16 ± 1.08 years, and an average age of 22 years and 4 months. All of the participants had completed the Higher Degree of interpretation at the Superior Conservatory of Music of Malaga (Spain). All subjects were healthy non-smokers, without any medical problems or medications that could have influenced their autonomic responses. All volunteers granted a valid written informed consent.

As a result of various circumstances encountered during the recording process, including movements artifacts, recording failures and signal loss, the cardiovascular parameters of 3 of the volunteers were unable to be calculated. The final number of thoroughly examined pianists was six.

ECG in DII, beat to beat arterial blood pressure and cutaneous cholinergic sympathetic response were continuously recorded during a piano concert. The work to be performed was composed in 1894 by Alexander Scriabin (1862–1915), written only for the left hand, and entitled: “Prelude et Nocturne op. 9 no 1,” written in C # Major and 3/4-time signature. The mean time of personal study of the work was 2.49 h ± 28 min.

The goal of selecting a piano piece written only for the left hand was to leave the right hand free to be used in data recording. By using the free right-hand, interferences were minimized in the recording of beat-to-beat blood pressure and cutaneous sympathetic activity. The measuring instruments were positioned in a way that caused minimal discomfort, creating minimal pressure on the fingers. Additionally, the arm rested on a support cushion that each pianist adjusted. The angle, height, lateralization, and even some movement could be customized as the cushion had wheels at its base. Due to these accommodations, the interference with the interpretation were minimal.

The following protocol was carried out.

1. Recording of autonomic parameters in sitting position during 5 min of relaxation in the absence of stimuli. We named this phase “Initial rest.”
2. Recording of autonomic parameters for 5 min in which the volunteers could freely play scales, arpeggios, thirds, trills, etc. … on the piano … We named this phase “Warm-up.”
3. Recording of autonomic parameters during a 5-min break. We named this phase “Pre-Concert Rest.”
4. Recording of autonomic parameters, during 5 min of interpretation of the piece, in the presence of professional pianists and professors. We named this phase “Concert.”

To standardize and establish a correlation between the changes documented in the recordings and the individual components comprising the musical composition, a consistent tempo of ∫ = 50 (Larghetto) was employed throughout the entire performance. To ensure temporal consistency, a metronome was initiated 30 s prior to the beginning of this phase.

5. Recording of autonomic parameters during 5 min of relaxation in the absence of stimuli. We named this phase “Post-Concert rest.”

### Variables

**Figure 1:**
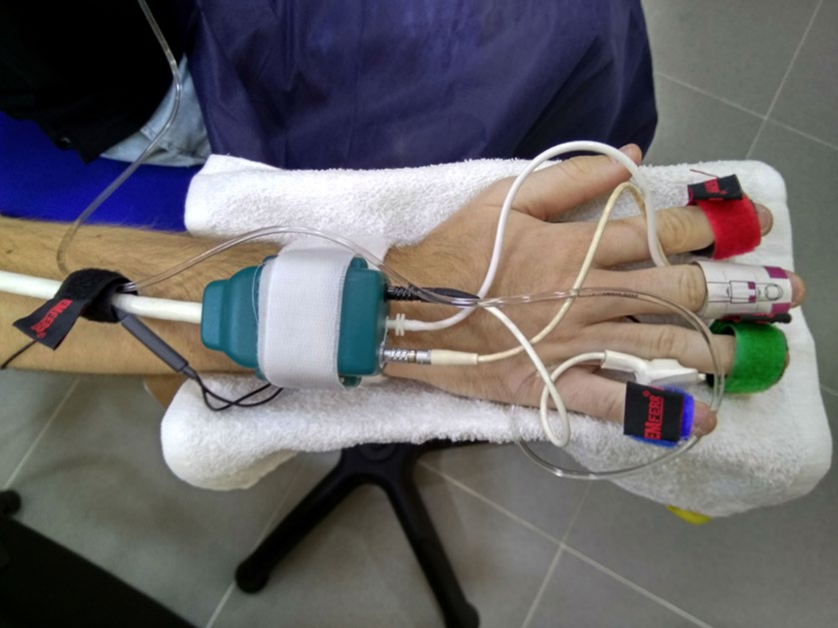
Recording system for cardiovascular parameters (middle and pinkie fingers) and cutaneous sympathetic activity (ring and index fingers) in the pianist’s right hand.

Continuous beat-to-beat non-invasive blood pressure recording was obtained with a Bmeye Nexfin® photoplethysmography system. The sensor cuff was located in the second phalanx of the middle finger of the right hand with a hydrostatic compensator in the little finger. The system was calibrated and contrasted with the data recorded using an OMROM M6 Comfort IT blood pressure monitor with PC connection (Figure 1).

ECG in DII (Cardioline-Delta 1 Plus®) was recorded with disposable adhesive button foam standard ECG Ag/AgCl electrodes. The recording of sympathetic cutaneous cholinergic activity was performed on the tip of the first phalanx of the ring and index fingers of the right hand with two non-polarizable Ag/AgCl electrodes (TSD203) through changes in skin conductance (GSR 100C amplifier) (Figure 1).

Signals were digitalized with the Biopac® MP160 CE multi modular system converter and analysed with the Acqknowledge 5.04 signal analysis software.

Mean heart rate, RRI, systolic blood pressure (SBP), diastolic blood pressure (DBP), mean blood pressure (MBP) and cutaneous conductance values were measured continuously during each 5 min.

In order to assess cardiac autonomic modulation, we performed Wavelet analysis using the Wigner-Ville transform (W-V) for time/frequency domain analysis with standard MatLab software. We determined sympathetic and parasympathetic modulation of RR intervals (RRI) in the low frequency (LF; 0.04–0.14 Hz) and high frequency (HF; 0.15–0.50 Hz) ranges (Task Force of the European Society of Cardiology the North A, 1996; Hilz and Dütsch, 2006; Li and Zheng, 2022). LF modulation of RRI reflects mainly sympathetic tone and, to an undetermined degree, also parasympathetic modulation; HF oscillations of RRIs reflect parasympathetic modulation (Task Force of the European Society of Cardiology the North A, 1996; Hilz and Dütsch, 2006; Li and Zheng, 2022). We also calculated the ratios between RRI modulations in the LF and HF ranges, using the LF/HF ratios as index of the sympathetic-parasympathetic balance (Task Force of the European Society of Cardiology the North A, 1996; Hilz and Dütsch, 2006; Li and Zheng, 2022). To reduce the parasympathetic component of LF modulation, we normalized LF and HF powers of RRI by calculating percentage values of LF and HF powers, with RRI LFnu = [LF/(LF + HF)] × 100%, and RRIHFnu = [HF/(LF + HF)] × 100% (Task Force of the European Society of Cardiology the North A, 1996; Hilz and Dütsch, 2006; Li and Zheng, 2022). The baroreceptor reflex sensitivity was assessed with the coherence-thresholder transfer function analysis method, computed from the Continuous Wavelet Transform (CWT) using the R package biwavelet.

Data recording and storage was obtained using 3 computers with Windows 10 operating system connected in a network.

The instrument of choice for musical study was a Yamaha P125 digital piano, and for performance was a Kawai CA-63 upright electric piano.

Throughout the process, volunteers were videotaped with a Logitech C-390 camera.

This study in no case can be considered as clinical experimentation in human beings. The ethical principles contained in the Declaration of Helsinki of the World Medical Association (2013) have been followed at all times. The volunteers granted a valid written informed consent and the protocols followed were approved by the Research Ethics Committee of the province of Málaga (Andalucía). Personal and clinical data are confidential and treated in accordance with the provisions of Regulation (EU) 2016/679 “General Data Protection” and Organic Law 3/2018, of December 5, on “Personal Data Protection.”

### Statistical analysis

Data processing was carried out using statistical package GraphPad Prism 9. Descriptive analysis of continuous quantitative variables was performed, obtaining mean, standard deviation, minimum and maximum for each parameter.

For analytical statistics, data were grouped into five comparison groups according to the moment of the study (Initial rest, Warm-up, Pre-Concert rest, Concert, Post-Concert rest). The significance level was established at *p* < 0.05. Repeated measures of one-way ANOVA with the Geisser–Greenhouse correction were used. Tukey multiple comparisons test, was used as a *post hoc* test for multiple comparisons after ANOVA (adjusted *p* values <0.05 were considered significant). In all cases, graphical representations in the form of box plot have been used.

## Results

### Heart rate

The mean heart rate during the “Initial rest” was 89.67 ± 4.1 bpm (from 70.56 bpm to 98.4 bpm), “Warm-up” 95.71 ± 3.56 bpm (from 85.2 to 107.11 bpm), “Pre-Concert rest” 90.75 ± 3.44 bpm (from 75.3 to 100.12 bpm), “Concert” 109.1 ± 3.85 bpm (from 94.27 to 120.91 bpm), and “Post-Concert rest” 90.20 ± 3.76 bpm (from 77.79 to 106.46 bpm).

The following significant changes in heart rate were obtained (Table 1):

**Table 1:** Changes in heart rate.

		Change	Tukey	
Initial rest	Concert	Increase	0.0040	*p* < 0.01
Warm-up	Concert	Increase	0.0430	*p* < 0.05
Pre-C-rest	Concert	Increase	0.0045	*p* < 0.01
Concert	Post-C-rest	Decrease	0.0014	*p* < 0.01

RM one-way ANOVA (*F* = 23.13; *p* < 0.0001), with Geisser–Greenhouse correction and Tukey’s multiple comparisons test (adjusted *p* value).

**Figure 2:**
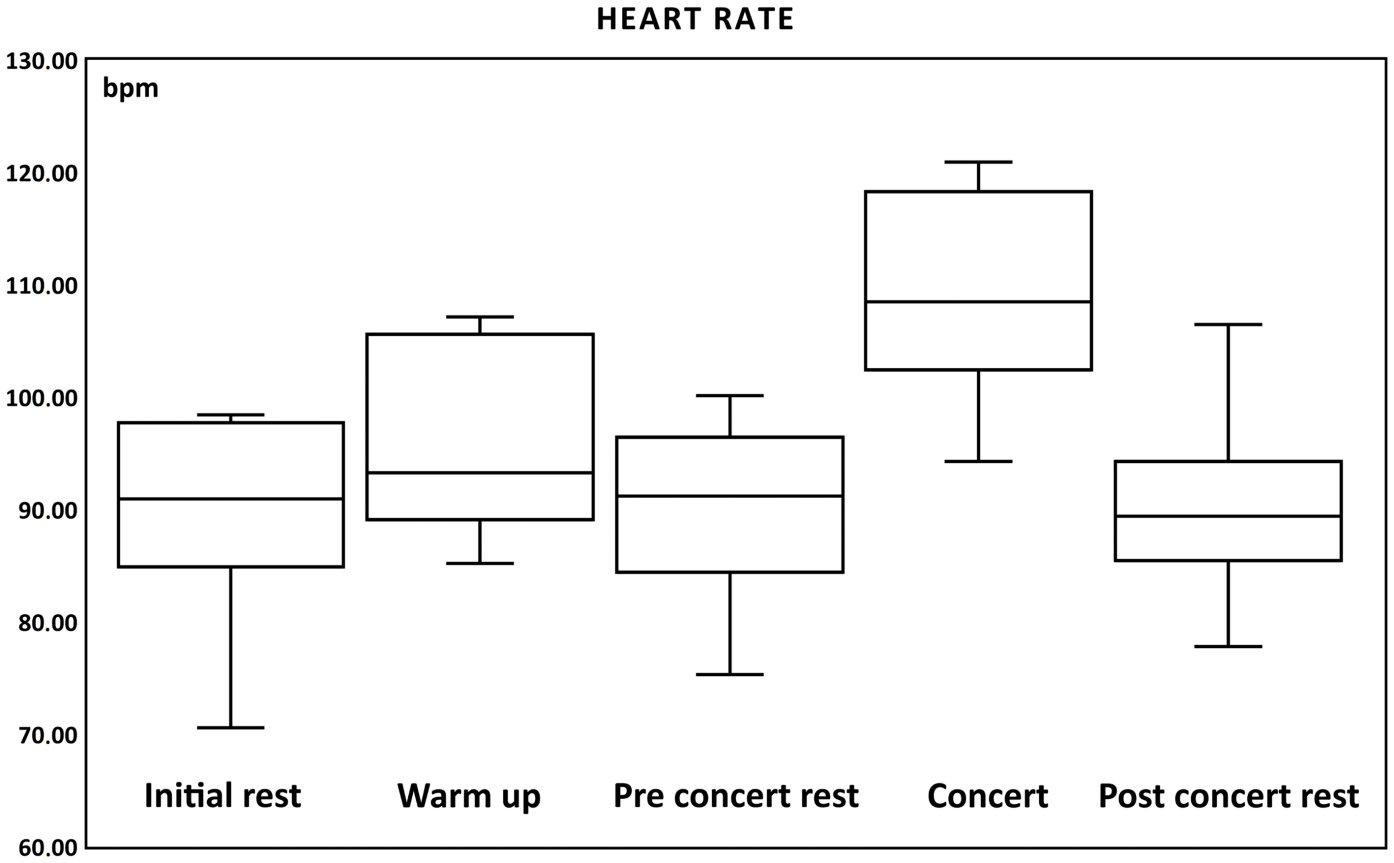
Box and whisker plots of changes of heart rate (bpm, beats per minute) during: initial rest, warm up, pre-concert rest, concert and post-concert rest.

The results show an increase in heart rate during the concert (Figure 2).

### Blood pressure

#### Systolic blood pressure

The systolic blood pressure during “Initial rest” was 122.17 ± 2.75 mmHg (from 109.24 to 128.33 mmHg), “Warm-up” 134.55 ± 3.72 mmHg (from 119.73 to 145.32 mmHg), “Pre-Concert rest” 121.55 ± 3.28 mmHg (from 108.34 to 130.78 mmHg), “Concert” 146.13 ± 5.11 mmHg (from 128.99 to 165.52 mmHg), and “Post-Concert rest” of 123.53 ± 5.61 mmHg (from 106.1 to 144.25 mmHg).

The following significant changes in systolic blood pressure were obtained (Table 2):

**Table 2:** Changes in systolic Blood pressure.

		Change	Tukey	
Initial rest	Warm up	Increase	0.0158	*p* < 0.05
Warm up	Pre-Concert	Decrease	0.0025	*p* < 0.01
Pre-concert	Concert	Increase	0.0009	*p* < 0.001
Concert	Post-Concert	Decrease	0.0004	*p* < 0.001
Warm up	Concert	Increase	0.0209	*p* < 0.05
Initial rest	Concert	Increase	0.0115	*p* < 0.05

RM one-way ANOVA (*F* = 25.21; *p* = 0.0010), with Geisser–Greenhouse correction and Tukey’s multiple comparisons test (adjusted *p* value).

**Figure 3:**
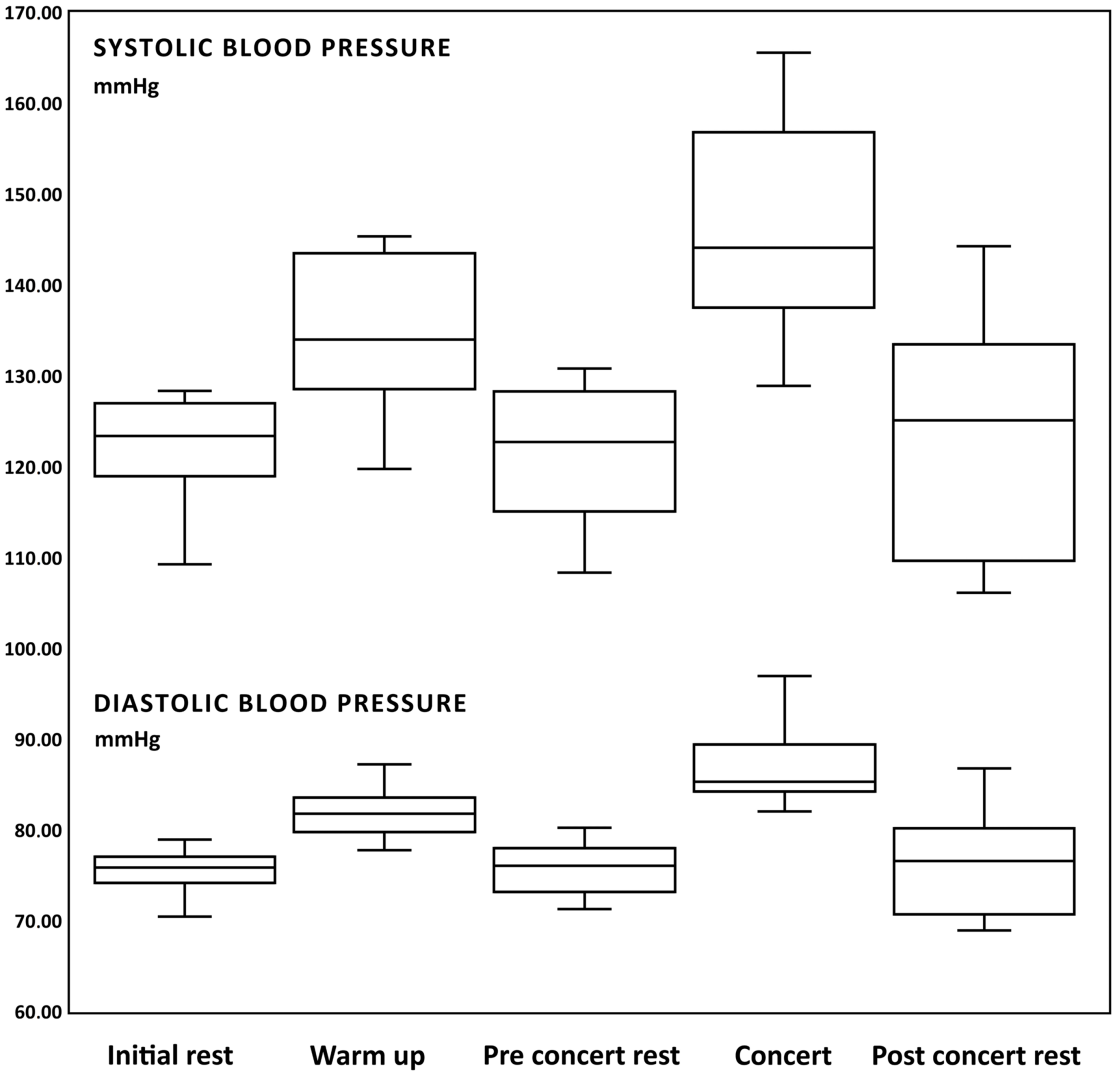
Box and whisker plots of changes of systolic and diastolic blood pressure (mmHg) during: initial rest, warm up, pre-concert rest, concert and post-concert rest.

The results show an increase in systolic pressure during the Warm-up and the Concert (Figure 3).

#### Diastolic blood pressure

The diastolic blood pressure during “Initial rest” was 75.47 ± 1.13 mmHg (from 70.43 to 78.92 mmHg), “Warm-up” 81.86 ± 1.26 mmHg (from 77.73 to 87.21 mmHg), “Pre-Concert rest” 75.74 ± 1.24 mmHg (from 71.26 to 80.2 mmHg), “concert” 86.89 ± 2.1 mmHg (from 82.01 to 96.92 mmHg), and “Post-Concert rest” 76.33 ± 2.53 mmHg (from 68.89 to 86.76 mmHg).

The following significant changes of diastolic blood pressure were obtained (Table 3):

**Table 3:** Changes in diastolic blood pressure.

		Change	Tukey	
Initial rest	Warm up	Increase	0.0145	*p* < 0.05
Warm up	Pre-Concert	Decrease	0.0013	*p* < 0.01
Pre-concert	Concert	Increase	0.0017	*p* < 0.01
Concert	Post-Concert	Decrease	0.0011	*p* < 0.001
Warm up	Concert	Increase	0.0192	*p* < 0.05
Initial rest	Concert	Increase	0.0124	*p* < 0.05

RM one-way ANOVA (*F* = 23.85; *p* = 0.0008), with Geisser–greenhouse correction and Tukey’s multiple comparisons test (adjusted *P* value).

The results show an increase in diastolic pressure during the Warm-up and the Concert (Figure 3).

### Cutaneous conductance (sympathetic cholinergic sudomotor activity)

The mean cutaneous conductance during “Initial rest” was 8.875.10^−2^ ± 8.10^−4^ μΩ, “Warm up” 8.833.10^−2^ ± 6.2.10^−4^ μΩ, “Pre-Concert rest” 8.797.10^−2^ ± 5.5.10^−4^ μΩ, “Concert” 9.341.10^−2^ ± 4.1.10^−3^ μΩ, and “Post-Concert rest” 8.852.10^−2^ ± 1.58.10^−3^ μΩ.

No significant differences were observed between groups.

However, conductance rose outside these predefined periods. This elevation coincides with the 30 s prior to the concert, and corresponds to the start of the metronome activity. We have named this period “Pre-Concert metronome.” The mean skin conductance values measured in this new period were the following: 1.684.10^−1^ ± 2.18.10^−2^ μΩ. Using these new data, the following significant differences appear (Table 4):

**Table 4:** Changes in cutaneous conductance.

		Change	Tukey	
Initial rest	metronome	Increase	<0.0001	*p* < 0.001
Warm up	metronome	Increase	<0.0001	*p* < 0.001
Pre-concert	metronome	Increase	<0.0001	*p* < 0.001
metronome	Concert	Decrease	<0.0001	*p* < 0.001
metronome	Post-Concert	Decrease	<0.0001	*p* < 0.001

RM one-way ANOVA (*F* = 14.01; *p* < 0.0001), with Geisser–Greenhouse correction and Tukey’s multiple comparisons test (adjusted *P* value).

The results show an increase in sympathetic cholinergic sudomotor activity at the start of the metronome activity before the beginning of the concert.

### Heart rate variability: wavelet analysis with Wigner-Ville

The variability values for sympathetic activity in power normalized units (LFnu) were in “Initial rest” 79.00 ± 5.43 nu, “Warm-up” 80.43 ± 2.72 nu, “Pre-Concert rest” 79.44 ± 2.72 nu, “Concert” 88.35 ± 3.29 nu, and “Post-Concert rest” 77.86 ± 2.67 nu.

The following significant changes were obtained (Table 5):

**Table 5:** Changes in sympathetic activity (LFnu).

		Change	Tukey	
Initial rest	Concert	Increase	0.0022	*p* < 0.01
Warm up	Concert	Increase	<0.0001	*p* < 0.0001
Pre-concert	Concert	Increase	0.0015	*p* < 0.01
Concert	Post-concert	Decrease	0.0045	*p* < 0.01

RM one-way ANOVA (*F* = 21.35; *p* = 0.0012), with Geisser–Greenhouse correction and Tukey’s multiple comparisons test (adjusted *P* value).

The results show an increase in sympathetic activity (LF) during the concert.

The variability values for the parasympathetic activity in power normalized units (HFnu) were in “Initial rest” 21.00 ± 5.43 nu, “Warm-up” 23.77 ± 2.72 nu, “Pre-Concert rest” 21.13 ± 4.53, “Concert” 11.79 ± 3.29 nu, and “Post-Concert rest” 22.14 ± 2.67 nu.

The following significant changes were obtained (Table 6):

**Table 6:** Changes in parasympathetic activity (HFnu).

		Change	Tukey	
Initial rest	Concert	Decrease	0.0022	*p* < 0.01
Warm up	Concert	Decrease	<0.0001	*p* < 0.0001
Pre-concert	Concert	Decrease	0.0015	*p* < 0.01
Concert	Post-Concert	Increase	0.0045	*p* < 0.01

RM one-way ANOVA (*F* = 21.36; *p* = 0.0012), with Geisser–Greenhouse correction and Tukey’s multiple comparisons test (adjusted *P* value).

The results show a decrease in parasympathetic activity (HF) during the concert.

The variability values for the sympathetic/parasympathetic ratio (LF/HF) at “Initial rest” were 4.10 ± 1.34, “Warm up” 3.27 ± 0.52, “Pre-Concert rest” 4.08 ± 1.41 “Concert” 8.39 ± 3.26, and “Post-Concert rest” 3.59 ± 0.61.

The following significant changes were obtained (Table 7):

**Table 7:** Changes in sympathetic/parasympathetic ratio (LF/HF).

		Change	Tukey	
Initial rest	Concert	Increase	0.0139	*p* < 0.05
Warm up	Concert	Increase	0.0183	*p* < 0.05
Pre-concert	Concert	Increase	0.0177	*p* < 0.05
Concert	Post-Concert	Decrease	0.0395	*p* < 0.05

RM one-way ANOVA (*F* = 21.09; *p* = 0.0029), with Geisser–Greenhouse correction and Tukey’s multiple comparisons test (adjusted *P* value).

**Figure 4:**
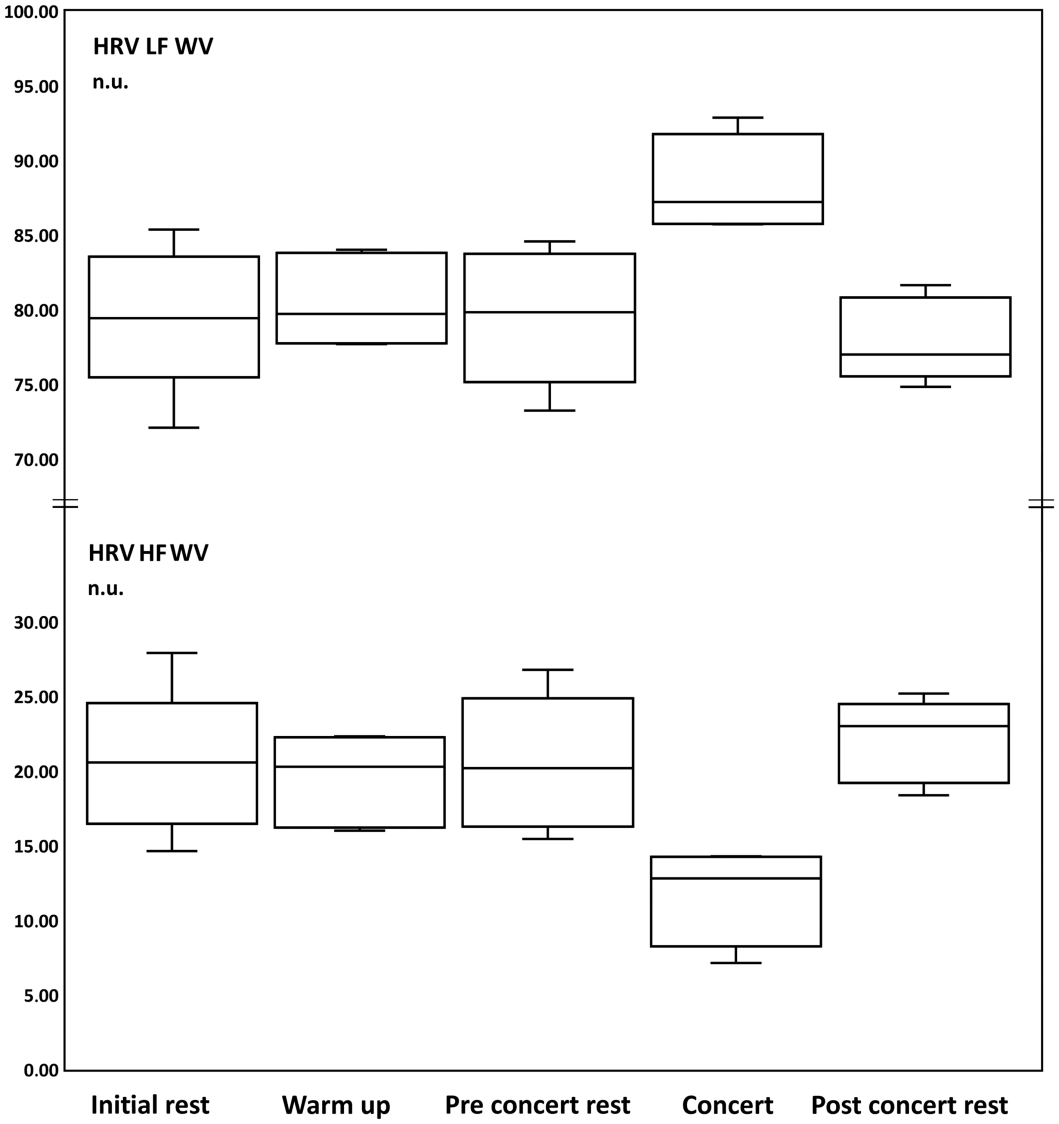
Box and whisker plots of changes of heart rate variability (HRV) calculated with Wigner-Ville analysis (W-V) of Low Frequency (LF, sympathetic activity) and High Frequency (HF, parasympathetic activity) in normalized units (n.u.) during: initial rest, warm up, pre-concert rest, concert and post-concert rest.

The results show that sympathetic activity was increased at rest. During the concert an increase of sympathetic activity (LFnu) with a decrease in parasympathetic activity (HFnu) is observed (Figure 4).

### Baroreceptor sensitivity

The vagal baroreceptor sensitivity values for LF at “Initial rest” were 8.83 ± 1.48 ms/mmHg (from 4.02 to 15.3 ms/mmHg), “Warm up” 6.77 ± 0.73 ms/mmHg (from 4.17 to 8.73 ms/mmHg), “Pre-Concert rest” 8.18 ± 1.25 ms/mmHg (from 4.09 to 13.41 ms/mmHg), “Concert” 5.65 ± 0.6 ms/mmHg (from 3.62 to 6.93 ms/mmHg), and “Post-Concert rest “7.9 ± 1.18 ms/mmHg (from 2.8 to 10.78 ms/mmHg).

**Figure 5:**
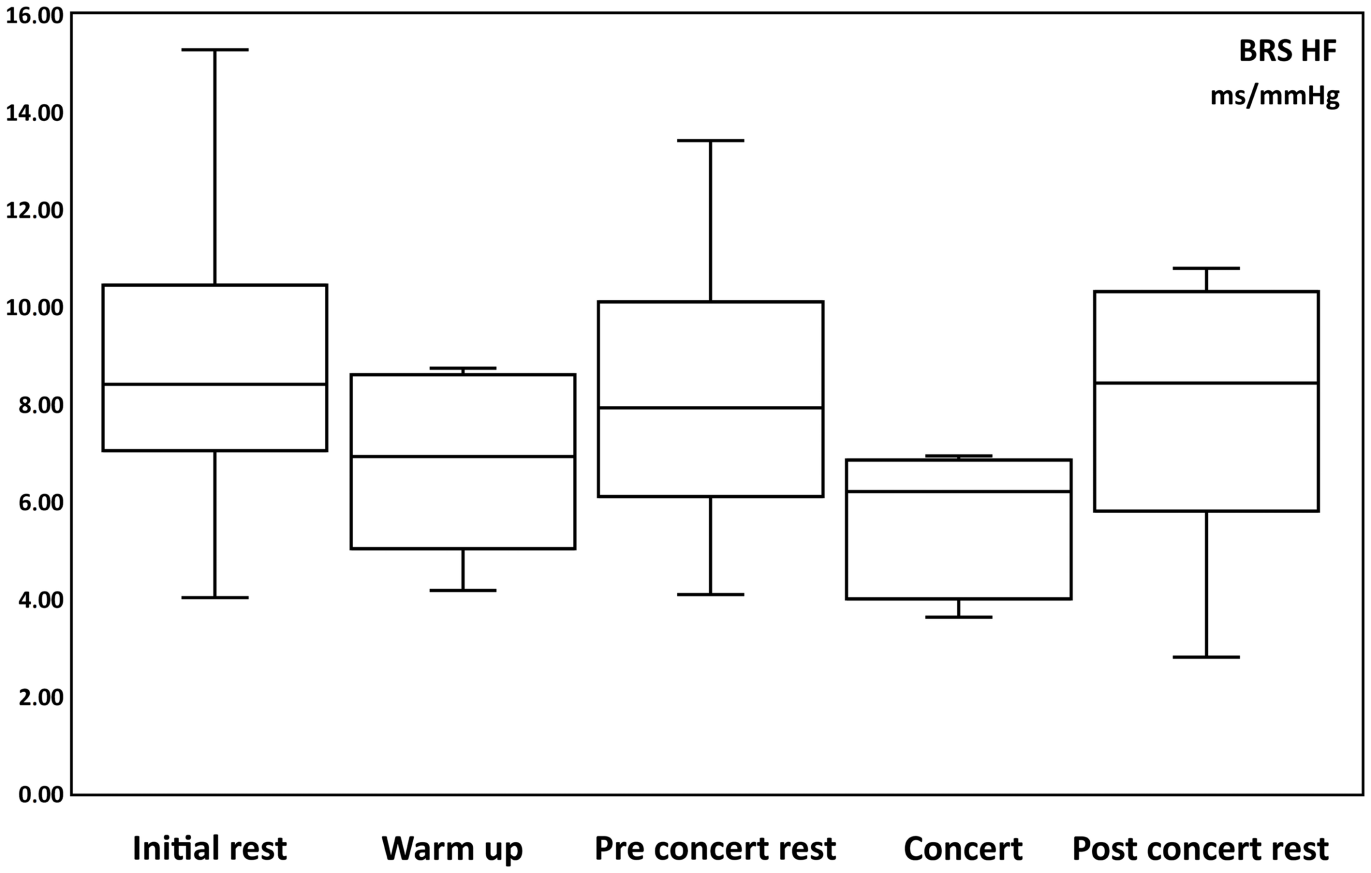
Box and whisker plots of changes of Baroreceptor Sensibility of High Frequency (BRS HF, parasympathetic activity) in ms/mmHg during: initial rest, warm up, pre-concert rest, concert and post-concert rest.

No changes were observed in vagal baroreceptor sensitivity during the concert (Figure 5).

The sympathetic baroreceptor sensitivity values for LF at “Initial rest” were 9.17 ± 1.32 ms/mmHg (from 4.75 to 14.8 ms/mmHg), “Warm up” 6.79 ± 1.06 ms/mmHg (from 4.14 to 11.57 ms/mmHg), “Pre-Concert rest” 7.46 ± 1.15 ms/mmHg (from 4.68 to 12.52 ms/mmHg), “Concert” 4.64 ± 0.77 ms/mmHg (from 2.8 to 8.1 ms/mmHg), and “Post-Concert rest “7.91 ± 1.31 ms/mmHg (from 3.15 to 13.01 ms/mmHg).

The following significant changes were obtained (Table 8):

**Table 8:** Changes in sympathetic baroreceptor sensibility.

		Change	Tukey	
Initial rest	Warm up	Decrease	0.0203	*p* < 0.05
Pre-concert	Concert	Decrease	0.0037	*p* < 0.01
Concert	Post-concert	Increase	0.0379	*p* < 0.05
Warm up	Concert	Decrease	0.0146	*p* < 0.05
Initial rest	Concert	Decrease	0.0045	*p* < 0.01

RM one-way ANOVA (*F* = 20.74; *p* = 0.0003), with Geisser–Greenhouse correction and Tukey’s multiple comparisons test (adjusted *P* value).

**Figure 6:**
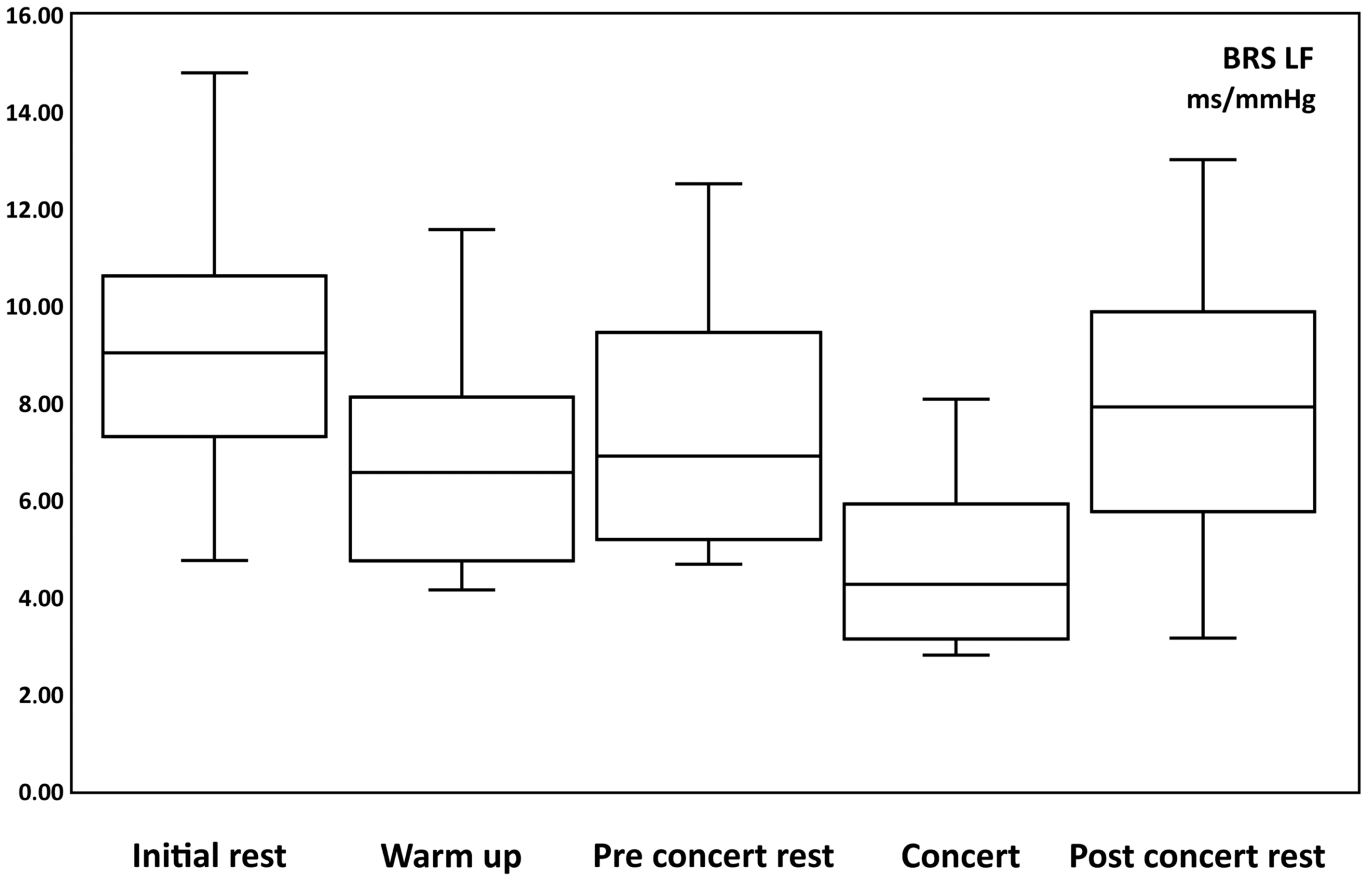
Box and whisker plots of changes of baroreceptor sensibility of low frequency (BRS LF, sympathetic activity) in ms/mmHg during: initial rest, warm up, pre-concert rest, concert and post-concert rest.

The results show a decrease in sympathetic baroreceptor sensitivity during concert. (Figure 6).

## Discussion

The objective of the present study was to elucidate blood pressure and autonomic responses that arise during and surrounding a musical performance, within a laboratory simulated live concert setting. We believe that the presence of other expert pianists, as well as, graduate and postgraduate piano teachers during the concert was a contributing factor in generating MPA.

This study shows that during a concert with a heightened level of stress there is a significant increase of heart rate, systolic, mean and diastolic arterial pressure. The W-V analysis shows a concomitant significant increase of cardiac adrenergic sympathetic flow and a decrease of parasympathetic flow together with a significant decrease in baroreceptor sensitivity. During Warm-up an increase in arterial blood pressure together with a decrease in sympathetic baroreceptor sensibility were also observed, although in a lesser extent. No changes were observed in sympathetic cholinergic sudomotor activity except for a short and transient increase just before the beginning of the concert, during a 30 s’s pre-metronomic phase.

### Methodological considerations

As previously expressed, we needed to select a work for piano with a non-operative right hand due to the presence of the recording sensors. Blood pressure was recorded by means of a pressure cuff placed on the second phalanx of the middle finger of the right hand. This circumstance was also used to record sympathetic skin conductance between the tips of the index and ring fingers of the right hand. We selected the “Prelude et Nocturne op. 9 no 1” written by Alexander Scriabin, who had chronic pain in the right hand and was therefore prone to writing works for the left hand (Altenmüller, 2015). There are other cases in which works are written for one hand with the aim of technically and expressively reinforce one of the two hands. In other cases, the objective is to be able to facilitate piano performance in situations in which a pianist loses the functionality of the hand due to an accident or a neuropathy (Boller and Bogousslavsky, 2015).

Initially, being all pianists right-handed, we supposed that a piece for the left hand could increase anxiety. However, one of the pillars of piano training is to develop ambidexterity in the performer, which is the ability to use both the right and left hand to play equally well, to tackle works that are part of the piano literature. The piece was chosen, in addition to leaving one hand free for data recording purposes, to be a work with technically undemanding interpretative characteristics for a professional pianist, so it should be performed with ease. Therefore, the impact of selecting the left hand for interpretation in the current study should have minimal effects. However, technically demanding pieces may be perceived with anxiety depending on the hand for which they were written. This is a question should be addressed in future studies.

### Heart rate changes during musical performance

The average heart rate increases during the concert and decreases again at final rest. No significant changes are found in warm up.

These changes in heart rate during musical performance are widely described in the literature (Yoshie et al., 2009a,b; Nakahara et al., 2010; Jha et al., 2022). Our results corroborate data previously published and reveal an increase in cardiac adrenergic sympathetic activity together with decrease in vagal activity that occurs during the performance of the piece in concert. The increase in heart rate is indicating the anxiety or stress to which the interpreter is subjected.

After the concert, at the final rest, cardiac sympathetic tone decreases and parasympathetic tone increases. During the Warm-up, we did not observe an increase in cardiac sympathetic or parasympathetic flow, which indicates that the interpreter is more relaxed.

### Changes in blood pressure during musical performance

To our knowledge this is the first study in which continuous beat-to-beat non-invasive blood pressure is recorded during a piano musical performance.

Diastolic and systolic blood pressures increase from rest to Warm-up, which is consistent with the need for increased blood flow to the structures involved in musical performance, the nervous and muscular systems.

During the concert, the increase in blood pressure is even greater, indicating an increase in the activity of the adrenergic sympathetic nervous system, probably secondary to the stress and anxiety of performing in front of an audience. This effect is observed both at the level of systolic pressure, which would indicate an increase in cardiac adrenergic sympathetic activity, and at the level of diastolic pressure, which indicates and increase in vascular tone and resistance due to an increase in peripheral adrenergic sympathetic activity.

After the concert, final rest, cardiac and peripheral sympathetic tone decreases to normal values.

The importance of this study lies in demonstrating for the first time what empirical knowledge pointed out during musical performance on blood pressure.

### Cholinergic sympathetic sudomotor activity/cutaneous conductance

It is striking that there were no significant differences in sudomotor activity in the four periods studied. As in previous papers (Nakahara et al., 2010), we were expecting an increase in sudomotor activity during the performance of the concert as a result of anxiety. However, and although not significantly, sweating decreases during the concert. It would be interesting to know if this decrease in sweating is due to the concentration of the interpreter on the work that he is executing at that moment. The increase in cutaneous cholinergic sympathetic activity during the preconcert metronomic phase is very significant. Cholinergic sympathetic activity is not modified at any other time and could be a useful indicator to assess the level of anxiety to which the interpreter is subjected before the performance of the work.

### Changes in heart rate variability and baroreceptor sensibility during musical performance

Once it was verified that during the concert there was a significant increase of heart rate and blood pressure, we analysed which changes in autonomic flow were responsible for these responses. To achieve this, we used the Wigner-Ville analysis, a technique based on Wavelet’s analysis, which analyses frequency changes in the time domain.

In musical performance, the changes in the recorded variables depend to a great extent on the emotional involvement and the technical difficulty of the elements that constitute a musical work. These changes are sometimes so fast that their effects cannot be detected by conventional methods of heart rate variability analysis Fast Fourier Transform (FFT) or Regression Analysis (AR). It is necessary to use a different technique that works not only in the frequency domain but also in the domain of the temporality of the work (Jha et al., 2022).

The Wigner-Ville analysis evaluates frequency changes in the time domain, demonstrating greater sensitivity to detect fast changes in cardiac sympathetic and parasympathetic activity variability in very short periods of time like in music performance. This analysis has been previously used to evaluate the variability of heart rate in non-musically trained listeners as a response to emotions induced by music, resulting in a suitable method of study (Orini et al., 2010).

At rest, we observed high values of sympathetic flow (74,05%) associated with low parasympathetic activity (26,91%), thus the sympathetic/parasympathetic relationship was bigger than 1, showing that volunteer musicians presented a certain degree of stress induced by the experimental conditions.

The W-V analysis also shows that during the concert the increase in heart rate and blood pressure is produced by an increase in cardiac adrenergic sympathetic tone together with a decrease in cardiac parasympathetic activity. To allow this increase in blood pressure, baroreceptor sensibility, which relates blood pressure and heart rate changes, decreases, thus resetting the baroreceptor response and allowing simultaneous increases of blood pressure and heart rate. This allows for adequate blood perfusion to meet the demand of active organs.

The results also show the appearance of an increase in cardiac adrenergic sympathetic activity during warm up. This increase in sympathetic tone is due to an increase in the demand for blood flow to the structures involved in musical performance, mainly brain and muscles of the left limb. During the concert, cardiac sympathetic flow increases even more, while parasympathetic activity decreases, indicating an elevated state of anxiety or stress in the interpreter. After the concert, during the post-concert rest, both cardiac sympathetic and parasympathetic tone returned to Initial rest values.

### General aspects

Our results are consistent with those of other studies reported in the literature. These authors (Yoshie et al., 2009a,b) found, with piano performances in stressful context, significant variations in the following parameters: increased heart rate and a greater electromyography magnitude in the muscles of the upper limb: biceps brachialis, common digital flexor of the fingers, biceps brachii and upper trapezius. However, they found an increase in the sweating rate in the concert situation (Yoshie et al., 2009a,b).

We also reinforce the studies that demonstrate emotional involvement in musical performance. These works show an increase in the heart rate and the sweating rate just at the beginning of the execution of the work. These responses are due to an increase in the activity of the sympathetic adrenergic and cholinergic nervous system, in addition, they found a decrease in parasympathetic tone (Nakahara et al., 2010).

Our work has shown to be effective in the quantitative and qualitative study of neurovegetative activation during musical performance. Judging from our study and previous studies in the literature, it appears that the degree of autonomic activation correlates with MPA levels (Yoshie et al., 2009a,b; Nakahara et al., 2010; Jha et al., 2022). The methodology employed could be used to categorise MPA by better distinguishing those subjects where MPA generates pathological responses and offering appropriate treatments for them. For example, musicians with focal dystonia are known to have higher levels of anxiety than musicians without focal dystonia, regardless of whether they are musicians or not. Taking this into consideration, treatment could be optimized according to the autonomic activation they generate since, as mentioned above, MPA increases motor tone in the upper extremity (Spahn, 2015).

Currently, there is a range of treatment options that can be offered for MPA: psychoanalytic or psychodynamic therapy, cognitive behavioral therapy, multimodal therapy, and in more refractory or severe cases, pharmacotherapy (Spahn, 2015; Fernholz et al., 2019).

Multimodal therapy is shown to be an effective treatment approach for MPA. This therapeutic approach is customized to meet the unique needs of each patient and combines in-depth psychodynamic insight into the individual’s prior performance experiences and their management, with cognitive-behavioural therapy to promote desensitization. The latter involves gradually exposing the musician to progressively more challenging and stressful performance scenarios (Guyon et al., 2022). The aim is to promote a positive sense of self-awareness by addressing experiences associated with MPA proactively instead of resorting to anxiety avoidance techniques (Spahn et al., 2021b).

Multimodal therapy integrates biofeedback of neurovegetative parameters to support the treatment. The easiest to use are heart rate and respiratory rate. More difficult, but possible, is to use blood pressure, skin conductance or muscle tone recording. This therapy is supported by exercises with the instrument or voice, as well as relaxation techniques to avoid the motor and neurovegetative response derived from MPA (Spahn et al., 2021a).

Lastly, in refractory or severe cases, beta-blockers may be used to reduce the neurovegetative responses generated by MPA. Nevertheless, it is common to rely on beta-blockers as the primary therapeutic intervention (Spahn, 2015). The use of beta-blockers represents an abortive treatment approach to alleviate anxiety symptoms. Since it does not address the underlying psychological factors that contribute to the problem, dependence and abuse can occur in long term use, in situations when the musician is required to perform under external pressure, such as in a concert or exam. For this reason, in most cases, they are contraindicated (Spahn et al., 2021a).

Finally, the prevention of MPA is important for the performer to develop without the limitations that this condition supposes. To achieve this, teachers should promote awareness of performance-related anxiety to their students from childhood and adolescence onwards and provide them with the necessary tools to address it from multiple perspectives in a personalized way (Spahn, 2015).

### Limitations

The main limitation in this study is the reduced number of participants due to the difficulty of finding professional pianists as volunteers, however, the sample size is comparable to other published studies. Another limitation is that the sample comprises only young subjects. We think it could be interesting to extend this study to healthy musicians of different age ranges and performing experience. It is well-known that autonomic responses decline with aging (Takla et al., 2023). Another limitation of this study is that the subjective assessment of the anxiety experienced by the performers was not evaluated, since it would have allowed to verify a possible correspondence between physiological changes and the perceived anxiety. Catecholamine plasma measurements could be of interest to asses objective anxiety and to solve pending issues regarding the physiological mechanisms involved in our findings (Yamasaki et al., 2021).

## Conclusion

To our knowledge, this study is the first to continuously analyse beat-to-beat blood pressure, demonstrating changes in blood pressure during musical performance. Additionally, this is the first time that the Wigner-Ville analysis is used to directly detect sympathetic and parasympathetic activity in MPA. We believe that this method of analysis, applied through the recording of instant heart rate frequency, could be an ideal method for working with MPA using biofeedback in multimodal therapy.

## Data availability statement

The raw data supporting the conclusions of this article will be made available by the authors, without undue reservation.

## Ethics statement

The studies involving human participants were reviewed and approved by the Research Ethics Committee of the province of Málaga (Andalucía, Spain). The patients/participants provided their written informed consent to participate in this study.

## Author contributions

JM-G, medical doctor and professional pianist, conducted all the experiments, data collection, data analysis and wrote the first manuscript draft and revisions. ML-G and CR made a significant contribution to data analysis and data interpretation, and revisions. AC-E contributed with Wigner Ville and continuous Wavelet transform analysis. MD-M designed the study, contributed to the data collection, data analysis, interpretation, and revisions to the manuscript. All authors contributed to the article and approved the submitted version.

## Funding

The study was supported by a program grant Junta de Andalucía, Group no. CTS-156, Spain. Part of the final study was supported with the Own Funds Program of the University of Málaga.

## Conflict of interest

The authors declare that the research was conducted in the absence of any commercial or financial relationships that could be construed as a potential conflict of interest.

## Publisher’s note

All claims expressed in this article are solely those of the authors and do not necessarily represent those of their affiliated organizations, or those of the publisher, the editors and the reviewers. Any product that may be evaluated in this article, or claim that may be made by its manufacturer, is not guaranteed or endorsed by the publisher.
